# 9-Benzyl-10-methyl­acridinium trifluoro­methane­sulfonate

**DOI:** 10.1107/S160053681001963X

**Published:** 2010-06-05

**Authors:** Damian Trzybiński, Beata Zadykowicz, Karol Krzymiński, Artur Sikorski, Jerzy Błażejowski

**Affiliations:** a Faculty of Chemistry, University of Gdańsk, J. Sobieskiego 18, 80-952 Gdańsk, Poland

## Abstract

In the crystal structure of the title compound, C_21_H_18_N^+^·CF_3_OS_3_^−^, the cations form inversion dimers through π–π inter­actions between the acridine ring systems. These dimers are further linked by C—H⋯π inter­actions. The cations and anions are connected by C—H⋯O, C—F⋯π and S—O⋯π inter­actions. The acridine and benzene ring systems are oriented at a dihedral angle of 76.8 (1)°with respect to each other. The acridine moieties are either parallel or inclined at an angle of 62.4 (1)° in the crystal structure.

## Related literature

For general background to acridinium derivatives, see: King *et al.* (2007 ▶); Roda *et al.* (2003 ▶); Wróblewska *et al.* (2004 ▶); Trzybiński *et al.* (2010 ▶); Zomer & Jacquemijns (2001 ▶). For related structures, see: Sikorski *et al.* (2007 ▶); Trzybiński *et al.* (2010 ▶). For inter­molecular inter­actions, see: Bianchi *et al.* (2004 ▶); Dorn *et al.* (2005 ▶); Hunter *et al.* (2001 ▶); Novoa *et al.* (2006 ▶); Takahashi *et al.* (2001 ▶). For the synthesis, see: Huntress & Shaw (1948 ▶); Sikorski *et al.* (2007 ▶); Trzybiński *et al.* (2010 ▶).
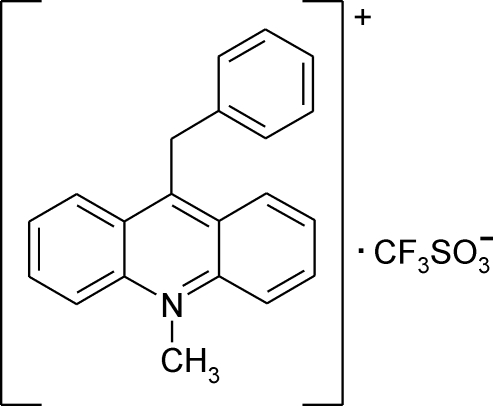


## Experimental

### 

#### Crystal data

C_21_H_18_N^+^·CF_3_O_3_S^−^
*M* *_r_* = 433.44
Monoclinic, 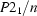

*a* = 14.6211 (7) Å
*b* = 8.2514 (2) Å
*c* = 17.2900 (8) Å
β = 107.707 (5)°
*V* = 1987.12 (15) Å^3^
*Z* = 4
Mo *K*α radiation
μ = 0.22 mm^−1^
*T* = 295 K
0.41 × 0.25 × 0.08 mm

#### Data collection

Oxford Diffraction Gemini R Ultra Ruby CCD diffractometer
Absorption correction: multi-scan (*CrysAlis RED*; Oxford Diffraction, 2008 ▶) *T*_min_ = 0.953, *T*_max_ = 0.988
16520 measured reflections
3528 independent reflections
2191 reflections with *I* > 2σ(*I*)
*R*_int_ = 0.048

#### Refinement

*R*[*F* ^2^ > 2σ(*F* ^2^)] = 0.052
*wR*(*F* ^2^) = 0.153
*S* = 1.06
3528 reflections
272 parameters
H-atom parameters constrained
Δρ_max_ = 0.37 e Å^−3^
Δρ_min_ = −0.28 e Å^−3^

### 

Data collection: *CrysAlis CCD* (Oxford Diffraction, 2008 ▶); cell refinement: *CrysAlis RED* (Oxford Diffraction, 2008 ▶); data reduction: *CrysAlis RED*; program(s) used to solve structure: *SHELXS97* (Sheldrick, 2008 ▶); program(s) used to refine structure: *SHELXL97* (Sheldrick, 2008 ▶); molecular graphics: *ORTEP-3* (Farrugia, 1997 ▶); software used to prepare material for publication: *SHELXL97* and *PLATON* (Spek, 2009 ▶).

## Supplementary Material

Crystal structure: contains datablocks global, I. DOI: 10.1107/S160053681001963X/om2343sup1.cif

Structure factors: contains datablocks I. DOI: 10.1107/S160053681001963X/om2343Isup2.hkl

Additional supplementary materials:  crystallographic information; 3D view; checkCIF report

## Figures and Tables

**Table 1:** Hydrogen-bond geometry (Å, °) *Cg*4 is the centroid of the C16–C21 ring.

*D*—H⋯*A*	*D*—H	H⋯*A*	*D*⋯*A*	*D*—H⋯*A*
C2—H2⋯O26^i^	0.93	2.49	3.398 (5)	167
C3—H3⋯*Cg*4^ii^	0.93	2.74	3.630 (5)	161
C15—H15*B*⋯O25^iii^	0.97	2.49	3.423 (4)	160
C22—H22*B*⋯O25^iv^	0.96	2.56	3.386 (5)	144
C22—H22*C*⋯O24	0.96	2.56	3.361 (5)	141

Symmetry codes: (i) 
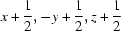
; (ii) 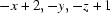; (iii) 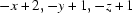; (iv) 
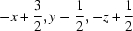
.

**Table 2:** C–F⋯π and S–O⋯π inter­actions (Å,°) *Cg*1 and *Cg*3 are the centroids of the C9/N10/C11–C14 and C5–C8/C13/C14 rings, respectively.

*X*	*I*	*J*	*I*⋯*J*	*X*⋯*J*	*X*–*I*⋯*J*
C27	F30	*Cg*3^v^	3.115 (3)	4.233 (3)	143.0 (2)
S23	O26	*Cg*1^v^	3.085 (3)	4.167 (2)	131.4 (2)

Symmetry code: (v) –*x* + 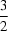, *y* + 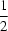, –*z* + 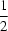.

**Table 3:** π–π inter­actions (Å, °) *Cg*1 and *Cg*2 are the centroids of the C9/N10/C11–C14 and C1–C4/C11/C12 rings, respectively. *CgI*⋯*CgJ* is the distance between ring centroids. The dihedral angle is that between the planes of the rings *I* and *J. CgI*_Perp is the perpendicular distance of *CgI* from ring *J. CgI*_Offset is the distance between *CgI* and perpendicular projection of *CgJ* on ring *I*.

*I*	*J*	*CgI*⋯*CgJ*	Dihedral angle	*CgI*_Perp	*CgI*_Offset
1	2^iii^	3.806 (2)	2.11 (15)	3.575 (2)	1.306 (2)
2	1^iii^	3.806 (2)	2.11 (15)	3.530 (2)	1.423 (2)
2	2^iii^	3.886 (2)	0.02 (15)	3.563 (2)	1.551 (2)

Symmetry code: (iii) –*x* + 2, –*y* + 1, –*z* + 1.

**Fig. 1.**
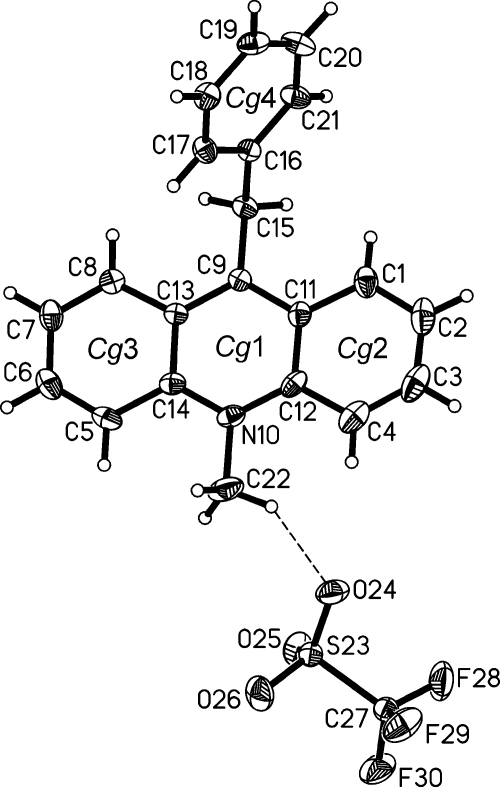
The molecular structure of the title compound showing the atom-labeling scheme. Displacement ellipsoids are drawn at the 25% probability level and H atoms are shown as small spheres of arbitrary radius. Cg1, Cg2, Cg3 and Cg4 denote the ring centroids. The C–H···O hydrogen bond is represented by a dashed line.

**Fig. 2.**
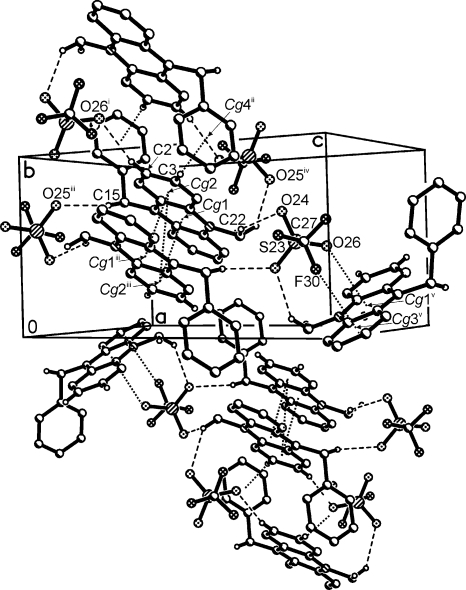
The arrangement of the ions in the crystal structure. The C–H···O interactions are represented by dashed lines, the C–H···π, C–F···π, S–O···π and π–π contacts by dotted lines. H atoms not involved in interactions have been omitted. [Symmetry codes: (i) x + 1/2, –y + 1/2, z + 1/2; (ii) –x + 2, –y, –z + 1; (iii) –x + 2, –y + 1, –z + 1; (iv) –x + 3/2, y – 1/2, –z + 1/2; (v) –x + 3/2, y + 1/2, –z + 1/2.]

## supplementary crystallographic information

### Comment

Quaternary  *N*-methylacridinium cations substituted in position 9 undergo oxidation with H_2_O_2_ or other oxidants in alkaline media accompanied by chemiluminescence (Zomer & Jacquemijns, 2001). The emission that originates from electronically excited 10-methyl-9-acridinone, the oxidation product, is affected by the features of the substituent in position 9. For these reasons various acridine derivatives of the above type have been synthesized and investigated from the point of view of their chemiluminogenic ability and applicability in immunological, biological, chemical and environmental analyses (Zomer & Jacquemijns, 2001; Roda *et al.*, 2003; King *et al.*, 2007). Continuing the search for 9-substituted acridinium derivatives with chemiluminogenic potential (Wróblewska *et al.*, 2004; Trzybiński *et al.*, 2010), we synthesized 9-benzyl-10-methylacridinium trifluoromethanesulfonate whose crystal structure is presented here.

In the crystal structure, the inversely oriented cations form dimers through multidirectional π–π interactions involving acridine moieties (Table 3, Fig. 2). These dimers are linked by C–H···O (Table 1, Figs. 1 and 2), C–F···π (acridine) (Table 2, Fig. 2) and S–O···π (acridine) (Table 2, Fig. 2) interactions with adjacent anions, and by C–H···π (phenyl) (Table 1, Fig. 2) interactions with neighboring cations. The C–H···O interactions are of the hydrogen bond type (Bianchi *et al.*, 2004; Novoa *et al.*, 2006). The C–H···π interactions should be of an attractive nature (Takahashi *et al.*, 2001), like the C–F···π (Dorn *et al.*, 2005), S–O···π (Dorn *et al.*, 2005) and the π–π (Hunter *et al.*, 2001) interactions. The crystal structure is stabilized by a network of these short-range specific interactions and by long-range electrostatic interactions between ions.

In the cation of the title compound (Fig. 1), the bond lengths and angles characterizing the geometry of the acridinium moiety are typical of acridine-based derivatives (Sikorski *et al.*, 2007; Trzybiński *et al.*, 2010). With respective average deviations from planarity of 0.0427 (3) Å and 0.0066 (3) Å, the acridine and benzene ring systems are oriented at 76.8 (1)°. The acridine moieties in pairs are parallel (remain at an angle of 0.0 (1)°), while in adjacent pairs they are inclined at an angle of 62.4 (1)°. The mutual arrangement of the acridine and benzene ring systems, as well as the acridine skeletons in the crystal lattice is similar in the compound investigated and its precursor – 9-benzylacridine (Sikorski *et al.*, 2007).

### Experimental

9-Benzylacridine was prepared by treating *N*-phenylaniline with an equimolar amount of phenylacetic acid, both dispersed in molten zinc chloride (Huntress & Shaw, 1948; Sikorski *et al.*, 2007). The crude product was purified chromatographically (SiO_2_, cyclohexane-ethyl acetate, 5:2 v/v). The compound thus obtained was quaternarized with a five-fold molar excess of methyltrifluoromethanesulfonate dissolved in anhydrous dichloromethane (Trzybiński *et al.*, 2010). The crude 9-benzyl-10-methylacridinium trifluoromethanesulfonate was dissolved in a small amount of ethanol, filtered, and precipitated with a 25 v/v excess of diethyl ether. Light-orange crystals suitable for X-Ray investigations were grown from absolute ethanol solution (m.p. 478–480 K).

### Refinement

H atoms were positioned geometrically, with C—H = 0.93 Å, 0.96 Å and 0.97 Å for the aromatic, methyl and methylene H atoms, respectively, and constrained to ride on their parent atoms with *U*_iso_(H) = *xU*_eq_(C), where *x* = 1.2 for the aromatic and *x* = 1.5 for the aliphatic H atoms.

### Crystal data

**Table:**

C_21_H_18_N^+^·CF_3_O_3_S^−^	*F*(000) = 896
*M**_r_* = 433.44	*D*_x_ = 1.449 Mg m^−^^3^
Monoclinic, *P*2_1_/*n*	Mo *K*α radiation, λ = 0.71073 Å
Hall symbol: -P 2yn	Cell parameters from 5587 reflections
*a* = 14.6211 (7) Å	θ = 3.2–29.2°
*b* = 8.2514 (2) Å	µ = 0.22 mm^−^^1^
*c* = 17.2900 (8) Å	*T* = 295 K
β = 107.707 (5)°	Plate, light-orange
*V* = 1987.12 (15) Å^3^	0.41 × 0.25 × 0.08 mm
*Z* = 4	

### Data collection

**Table:**

Oxford Diffraction Gemini R Ultra Ruby CCD diffractometer	3528 independent reflections
Radiation source: Enhanced (Mo) X-ray Source	2191 reflections with *I* > 2σ(*I*)
graphite	*R*_int_ = 0.048
Detector resolution: 10.4002 pixels mm^-1^	θ_max_ = 25.1°, θ_min_ = 3.3°
ω scans	*h* = −17→12
Absorption correction: multi-scan (*CrysAlis RED*; Oxford Diffraction, 2008)	*k* = −9→9
*T*_min_ = 0.953, *T*_max_ = 0.988	*l* = −20→20
16520 measured reflections	

### Refinement

**Table:**

Refinement on *F*^2^	Primary atom site location: structure-invariant direct methods
Least-squares matrix: full	Secondary atom site location: difference Fourier map
*R*[*F*^2^ > 2σ(*F*^2^)] = 0.052	Hydrogen site location: inferred from neighbouring sites
*wR*(*F*^2^) = 0.153	H-atom parameters constrained
*S* = 1.06	*w* = 1/[σ^2^(*F*_o_^2^) + (0.0814*P*)^2^ + 0.218*P*] where *P* = (*F*_o_^2^ + 2*F*_c_^2^)/3
3528 reflections	(Δ/σ)_max_ = 0.001
272 parameters	Δρ_max_ = 0.37 e Å^−^^3^
0 restraints	Δρ_min_ = −0.28 e Å^−^^3^

### Special details

**Table:**

Geometry. All esds (except the esd in the dihedral angle between two l.s. planes) are estimated using the full covariance matrix. The cell esds are taken into account individually in the estimation of esds in distances, angles and torsion angles; correlations between esds in cell parameters are only used when they are defined by crystal symmetry. An approximate (isotropic) treatment of cell esds is used for estimating esds involving l.s. planes.

### Fractional atomic coordinates and isotropic or equivalent isotropic displacement parameters (Å^2^)

**Table:**

	*x*	*y*	*z*	*U*_iso_*/*U*_eq_	
C1	1.0142 (2)	0.2164 (4)	0.5705 (2)	0.0680 (10)	
H1	1.0341	0.1838	0.6245	0.082*	
C2	1.0692 (3)	0.1812 (4)	0.5215 (3)	0.0870 (13)	
H2	1.1267	0.1251	0.5419	0.104*	
C3	1.0384 (4)	0.2302 (5)	0.4410 (3)	0.0919 (14)	
H3	1.0760	0.2037	0.4081	0.110*	
C4	0.9568 (3)	0.3141 (4)	0.4080 (3)	0.0793 (11)	
H4	0.9395	0.3451	0.3538	0.095*	
C5	0.6717 (3)	0.5685 (3)	0.4389 (2)	0.0616 (9)	
H5	0.6566	0.6103	0.3866	0.074*	
C6	0.6130 (3)	0.5955 (4)	0.4840 (3)	0.0703 (10)	
H6	0.5572	0.6554	0.4620	0.084*	
C7	0.6331 (2)	0.5369 (4)	0.5617 (2)	0.0641 (9)	
H7	0.5903	0.5551	0.5911	0.077*	
C8	0.7146 (2)	0.4531 (3)	0.59546 (18)	0.0515 (8)	
H8	0.7275	0.4155	0.6484	0.062*	
C9	0.8675 (2)	0.3374 (3)	0.58718 (16)	0.0416 (7)	
N10	0.8163 (2)	0.4426 (3)	0.42625 (14)	0.0545 (7)	
C11	0.9266 (2)	0.3027 (3)	0.53895 (18)	0.0490 (8)	
C12	0.8982 (2)	0.3545 (3)	0.45600 (19)	0.0536 (8)	
C13	0.7815 (2)	0.4203 (3)	0.55233 (15)	0.0411 (7)	
C14	0.7575 (2)	0.4760 (3)	0.47112 (18)	0.0486 (8)	
C15	0.8974 (2)	0.2879 (3)	0.67532 (17)	0.0514 (8)	
H15A	0.8641	0.3559	0.7039	0.062*	
H15B	0.9657	0.3076	0.6987	0.062*	
C16	0.8769 (2)	0.1104 (3)	0.68912 (16)	0.0450 (7)	
C17	0.7896 (2)	0.0396 (3)	0.64921 (19)	0.0565 (8)	
H17	0.7431	0.0991	0.6111	0.068*	
C18	0.7708 (2)	−0.1185 (4)	0.6653 (2)	0.0623 (9)	
H18	0.7117	−0.1645	0.6383	0.075*	
C19	0.8388 (3)	−0.2080 (4)	0.72059 (19)	0.0661 (10)	
H19	0.8256	−0.3141	0.7320	0.079*	
C20	0.9259 (3)	−0.1408 (4)	0.7590 (2)	0.0739 (11)	
H20	0.9730	−0.2022	0.7954	0.089*	
C21	0.9445 (3)	0.0179 (4)	0.74392 (18)	0.0627 (9)	
H21	1.0038	0.0631	0.7713	0.075*	
C22	0.7881 (3)	0.4991 (6)	0.3401 (2)	0.0921 (13)	
H22A	0.7789	0.6144	0.3383	0.138*	
H22B	0.7294	0.4470	0.3098	0.138*	
H22C	0.8379	0.4721	0.3167	0.138*	
S23	0.84575 (6)	0.55745 (8)	0.14455 (5)	0.0509 (3)	
O24	0.8608 (2)	0.4008 (3)	0.17948 (15)	0.0869 (8)	
O25	0.87049 (18)	0.6881 (3)	0.20097 (14)	0.0771 (7)	
O26	0.75711 (18)	0.5802 (3)	0.08205 (16)	0.0925 (8)	
C27	0.9326 (2)	0.5715 (3)	0.09040 (19)	0.0553 (8)	
F28	1.01928 (18)	0.5412 (4)	0.13525 (16)	0.1322 (11)	
F29	0.9156 (2)	0.4660 (3)	0.03015 (15)	0.1090 (9)	
F30	0.9335 (2)	0.7119 (3)	0.05685 (18)	0.1184 (9)	

### Atomic displacement parameters (Å^2^)

**Table:**

	*U*^11^	*U*^22^	*U*^33^	*U*^12^	*U*^13^	*U*^23^
C1	0.051 (2)	0.0523 (17)	0.102 (3)	−0.0032 (16)	0.025 (2)	−0.0051 (18)
C2	0.062 (3)	0.061 (2)	0.147 (4)	−0.0002 (18)	0.045 (3)	−0.014 (3)
C3	0.098 (4)	0.074 (3)	0.133 (4)	−0.020 (2)	0.078 (3)	−0.035 (3)
C4	0.094 (3)	0.070 (2)	0.092 (3)	−0.021 (2)	0.055 (3)	−0.021 (2)
C5	0.063 (2)	0.0494 (17)	0.058 (2)	−0.0072 (16)	−0.0032 (18)	0.0132 (15)
C6	0.058 (2)	0.0511 (18)	0.092 (3)	0.0070 (16)	0.007 (2)	−0.0005 (19)
C7	0.055 (2)	0.0556 (18)	0.082 (3)	0.0044 (16)	0.0208 (19)	−0.0152 (18)
C8	0.057 (2)	0.0479 (16)	0.0494 (17)	−0.0042 (15)	0.0155 (16)	−0.0066 (13)
C9	0.0406 (17)	0.0361 (14)	0.0437 (16)	−0.0092 (12)	0.0063 (14)	−0.0046 (12)
N10	0.0601 (18)	0.0573 (14)	0.0459 (14)	−0.0176 (13)	0.0158 (13)	0.0010 (12)
C11	0.0440 (18)	0.0397 (14)	0.065 (2)	−0.0079 (13)	0.0191 (16)	−0.0050 (14)
C12	0.061 (2)	0.0460 (16)	0.064 (2)	−0.0236 (15)	0.0350 (18)	−0.0155 (15)
C13	0.0463 (17)	0.0345 (13)	0.0397 (15)	−0.0065 (12)	0.0091 (13)	−0.0031 (12)
C14	0.054 (2)	0.0409 (15)	0.0485 (18)	−0.0132 (13)	0.0121 (16)	0.0005 (13)
C15	0.0536 (19)	0.0475 (16)	0.0480 (17)	−0.0013 (13)	0.0078 (15)	0.0024 (13)
C16	0.0483 (18)	0.0450 (15)	0.0387 (15)	−0.0019 (13)	0.0088 (14)	−0.0037 (12)
C17	0.0489 (19)	0.0520 (17)	0.0626 (19)	−0.0003 (14)	0.0081 (16)	−0.0030 (15)
C18	0.061 (2)	0.0570 (19)	0.069 (2)	−0.0097 (16)	0.0209 (18)	−0.0068 (17)
C19	0.101 (3)	0.0482 (17)	0.055 (2)	−0.0102 (19)	0.032 (2)	−0.0009 (16)
C20	0.095 (3)	0.058 (2)	0.053 (2)	0.004 (2)	−0.0001 (19)	0.0128 (16)
C21	0.068 (2)	0.0598 (19)	0.0486 (18)	−0.0066 (16)	0.0000 (17)	0.0059 (15)
C22	0.095 (3)	0.134 (3)	0.046 (2)	−0.025 (3)	0.020 (2)	0.017 (2)
S23	0.0509 (5)	0.0466 (4)	0.0560 (5)	−0.0018 (3)	0.0174 (4)	−0.0017 (3)
O24	0.131 (2)	0.0614 (14)	0.0900 (17)	0.0137 (14)	0.0658 (17)	0.0192 (13)
O25	0.0813 (18)	0.0706 (14)	0.0855 (16)	−0.0086 (12)	0.0343 (14)	−0.0294 (13)
O26	0.0492 (15)	0.114 (2)	0.1010 (19)	−0.0060 (14)	0.0025 (14)	−0.0007 (16)
C27	0.058 (2)	0.0492 (17)	0.060 (2)	0.0005 (15)	0.0213 (17)	0.0023 (15)
F28	0.0559 (15)	0.234 (3)	0.1091 (19)	0.0300 (17)	0.0292 (14)	0.015 (2)
F29	0.159 (3)	0.0953 (15)	0.1012 (17)	−0.0266 (15)	0.0826 (17)	−0.0321 (13)
F30	0.156 (2)	0.0668 (13)	0.172 (2)	0.0008 (14)	0.108 (2)	0.0329 (14)

### Geometric parameters (Å, °)

**Table:**

C1—C2	1.364 (5)	C13—C14	1.416 (4)
C1—C11	1.421 (4)	C15—C16	1.528 (4)
C1—H1	0.9300	C15—H15A	0.9700
C2—C3	1.387 (6)	C15—H15B	0.9700
C2—H2	0.9300	C16—C21	1.374 (4)
C3—C4	1.346 (6)	C16—C17	1.382 (4)
C3—H3	0.9300	C17—C18	1.378 (4)
C4—C12	1.403 (5)	C17—H17	0.9300
C4—H4	0.9300	C18—C19	1.367 (4)
C5—C6	1.342 (5)	C18—H18	0.9300
C5—C14	1.428 (4)	C19—C20	1.363 (5)
C5—H5	0.9300	C19—H19	0.9300
C6—C7	1.372 (5)	C20—C21	1.378 (4)
C6—H6	0.9300	C20—H20	0.9300
C7—C8	1.348 (4)	C21—H21	0.9300
C7—H7	0.9300	C22—H22A	0.9600
C8—C13	1.425 (4)	C22—H22B	0.9600
C8—H8	0.9300	C22—H22C	0.9600
C9—C13	1.396 (4)	S23—O24	1.415 (2)
C9—C11	1.402 (4)	S23—O25	1.425 (2)
C9—C15	1.508 (4)	S23—O26	1.425 (2)
N10—C14	1.350 (4)	S23—C27	1.796 (3)
N10—C12	1.361 (4)	C27—F28	1.293 (4)
N10—C22	1.495 (4)	C27—F30	1.297 (3)
C11—C12	1.432 (4)	C27—F29	1.322 (3)
			
C2—C1—C11	120.1 (4)	C9—C15—C16	114.0 (2)
C2—C1—H1	120.0	C9—C15—H15A	108.8
C11—C1—H1	120.0	C16—C15—H15A	108.8
C1—C2—C3	119.2 (4)	C9—C15—H15B	108.8
C1—C2—H2	120.4	C16—C15—H15B	108.8
C3—C2—H2	120.4	H15A—C15—H15B	107.7
C4—C3—C2	123.4 (4)	C21—C16—C17	118.1 (3)
C4—C3—H3	118.3	C21—C16—C15	120.4 (3)
C2—C3—H3	118.3	C17—C16—C15	121.5 (2)
C3—C4—C12	119.6 (4)	C18—C17—C16	120.6 (3)
C3—C4—H4	120.2	C18—C17—H17	119.7
C12—C4—H4	120.2	C16—C17—H17	119.7
C6—C5—C14	120.2 (3)	C19—C18—C17	120.4 (3)
C6—C5—H5	119.9	C19—C18—H18	119.8
C14—C5—H5	119.9	C17—C18—H18	119.8
C5—C6—C7	121.7 (3)	C20—C19—C18	119.6 (3)
C5—C6—H6	119.2	C20—C19—H19	120.2
C7—C6—H6	119.2	C18—C19—H19	120.2
C8—C7—C6	120.2 (4)	C19—C20—C21	120.3 (3)
C8—C7—H7	119.9	C19—C20—H20	119.9
C6—C7—H7	119.9	C21—C20—H20	119.9
C7—C8—C13	121.9 (3)	C16—C21—C20	121.1 (3)
C7—C8—H8	119.1	C16—C21—H21	119.5
C13—C8—H8	119.1	C20—C21—H21	119.5
C13—C9—C11	118.7 (3)	N10—C22—H22A	109.5
C13—C9—C15	121.0 (3)	N10—C22—H22B	109.5
C11—C9—C15	120.2 (3)	H22A—C22—H22B	109.5
C14—N10—C12	122.2 (3)	N10—C22—H22C	109.5
C14—N10—C22	118.6 (3)	H22A—C22—H22C	109.5
C12—N10—C22	119.2 (3)	H22B—C22—H22C	109.5
C9—C11—C1	121.4 (3)	O24—S23—O25	115.13 (15)
C9—C11—C12	119.4 (3)	O24—S23—O26	115.57 (17)
C1—C11—C12	119.2 (3)	O25—S23—O26	113.74 (15)
N10—C12—C4	122.0 (3)	O24—S23—C27	103.79 (14)
N10—C12—C11	119.5 (3)	O25—S23—C27	103.62 (15)
C4—C12—C11	118.5 (3)	O26—S23—C27	102.73 (16)
C9—C13—C14	120.4 (3)	F28—C27—F30	107.4 (3)
C9—C13—C8	122.6 (2)	F28—C27—F29	105.1 (3)
C14—C13—C8	117.0 (3)	F30—C27—F29	105.1 (3)
N10—C14—C13	119.6 (3)	F28—C27—S23	113.2 (2)
N10—C14—C5	121.5 (3)	F30—C27—S23	113.3 (2)
C13—C14—C5	118.9 (3)	F29—C27—S23	112.0 (2)
			
C11—C1—C2—C3	−0.2 (5)	C12—N10—C14—C5	−179.9 (2)
C1—C2—C3—C4	1.2 (6)	C22—N10—C14—C5	−2.4 (4)
C2—C3—C4—C12	−0.6 (6)	C9—C13—C14—N10	2.8 (4)
C14—C5—C6—C7	0.4 (5)	C8—C13—C14—N10	−177.3 (2)
C5—C6—C7—C8	1.6 (5)	C9—C13—C14—C5	−176.4 (2)
C6—C7—C8—C13	−0.9 (4)	C8—C13—C14—C5	3.5 (4)
C13—C9—C11—C1	−179.0 (2)	C6—C5—C14—N10	177.8 (3)
C15—C9—C11—C1	2.0 (4)	C6—C5—C14—C13	−3.0 (4)
C13—C9—C11—C12	0.7 (4)	C13—C9—C15—C16	99.8 (3)
C15—C9—C11—C12	−178.3 (2)	C11—C9—C15—C16	−81.1 (3)
C2—C1—C11—C9	178.5 (3)	C9—C15—C16—C21	136.1 (3)
C2—C1—C11—C12	−1.2 (4)	C9—C15—C16—C17	−45.8 (4)
C14—N10—C12—C4	177.2 (3)	C21—C16—C17—C18	1.1 (5)
C22—N10—C12—C4	−0.4 (4)	C15—C16—C17—C18	−177.0 (3)
C14—N10—C12—C11	−3.7 (4)	C16—C17—C18—C19	−0.5 (5)
C22—N10—C12—C11	178.8 (3)	C17—C18—C19—C20	−1.0 (5)
C3—C4—C12—N10	178.3 (3)	C18—C19—C20—C21	1.9 (5)
C3—C4—C12—C11	−0.9 (5)	C17—C16—C21—C20	−0.2 (5)
C9—C11—C12—N10	2.8 (4)	C15—C16—C21—C20	177.9 (3)
C1—C11—C12—N10	−177.5 (2)	C19—C20—C21—C16	−1.3 (5)
C9—C11—C12—C4	−178.0 (3)	O24—S23—C27—F28	−54.1 (3)
C1—C11—C12—C4	1.7 (4)	O25—S23—C27—F28	66.5 (3)
C11—C9—C13—C14	−3.5 (4)	O26—S23—C27—F28	−174.8 (3)
C15—C9—C13—C14	175.5 (2)	O24—S23—C27—F30	−176.8 (3)
C11—C9—C13—C8	176.6 (2)	O25—S23—C27—F30	−56.1 (3)
C15—C9—C13—C8	−4.4 (4)	O26—S23—C27—F30	62.5 (3)
C7—C8—C13—C9	178.3 (2)	O24—S23—C27—F29	64.6 (3)
C7—C8—C13—C14	−1.7 (4)	O25—S23—C27—F29	−174.8 (2)
C12—N10—C14—C13	0.9 (4)	O26—S23—C27—F29	−56.2 (2)
C22—N10—C14—C13	178.5 (3)		

### Hydrogen-bond geometry (Å, °)

**Table:**

*Cg*4 is the centroid of the C16–C21 ring.

**Table:**

*D*—H···*A*	*D*—H	H···*A*	*D*···*A*	*D*—H···*A*
C2—H2···O26^i^	0.93	2.49	3.398 (5)	167
C3—H3···*Cg*4^ii^	0.93	2.74	3.630 (5)	161
C15—H15*B*···O25^iii^	0.97	2.49	3.423 (4)	160
C22—H22*B*···O25^iv^	0.96	2.56	3.386 (5)	144
C22—H22*C*···O24	0.96	2.56	3.361 (5)	141

Symmetry codes:  (i) *x*+1/2, −*y*+1/2, *z*+1/2; (ii) −*x*+2, −*y*, −*z*+1; (iii) −*x*+2, −*y*+1, −*z*+1; (iv) −*x*+3/2, *y*−1/2, −*z*+1/2.

### Table 2 C–F···π and S–O···π interactions (Å,°)

*Cg*1 and *Cg*3 are the centroids of the C9/N10/C11–C14 and C5–C8/C13/C14 rings, respectively.

**Table:**

*X*	*I*	*J*	*I*···*J*	*X*···*J*	*X*–*I*···*J*
C27	F30	*Cg*3^v^	3.115 (3)	4.233 (3)	143.0 (2)
S23	O26	*Cg*1^v^	3.085 (3)	4.167 (2)	131.4 (2)

Symmetry code: (v) –*x* + 3/2, *y* + 1/2, –*z* + 1/2.

### Table 3 π–π interactions (Å, °)

*Cg*1 and *Cg*2 are the centroids of the C9/N10/C11–C14 and C1–C4/C11/C12 rings, respectively. *CgI*···*CgJ* is the distance between ring centroids. The dihedral angle is that between the planes of the rings *I* and *J*. *CgI*_Perp is the perpendicular distance of *CgI* from ring *J*. Cg*I*_Offset is the distance between *CgI* and perpendicular projection of *CgJ* on ring *I*.

**Table:**

*I*	*J*	*CgI*···*CgJ*	Dihedral angle	*CgI*_Perp	*CgI*_Offset
1	2^iii^	3.806 (2)	2.11 (15)	3.575 (2)	1.306 (2)
2	1^iii^	3.806 (2)	2.11 (15)	3.530 (2)	1.423 (2)
2	2^iii^	3.886 (2)	0.02 (15)	3.563 (2)	1.551 (2)

Symmetry code: (iii) –*x* + 2, –*y* + 1, –*z* + 1.
